# Liver Failure Impairs the Intrahepatic Elimination of Interleukin-6, Tumor Necrosis Factor-Alpha, Hepatocyte Growth Factor, and Transforming Growth Factor-Beta

**DOI:** 10.1155/2015/934065

**Published:** 2015-05-21

**Authors:** Dawid Porowski, Agnieszka Wirkowska, Ewa Hryniewiecka, Janusz Wyzgał, Marek Pacholczyk, Leszek Pączek

**Affiliations:** ^1^Transplantation Institute, Department of Immunology, Transplantology and Internal Diseases, Medical University of Warsaw, 59 Nowogrodzka Street, 02-006 Warsaw, Poland; ^2^Department of Nephrological Nursing, Medical University of Warsaw, 8 Oczki Street, 02-007 Warsaw, Poland; ^3^Transplantation Institute, Department of General and Transplant Surgery, Medical University of Warsaw, 59 Nowogrodzka Street, 02-006 Warsaw, Poland

## Abstract

The strategic location of the liver and its metabolic activity make it a key organ regulating homeostasis. Our purpose was to examine its participation in removal of cytokines: interleukin-6 (Il-6), tumor necrosis factor-alpha (TNF-*α*), hepatocyte growth factor (HGF), and transforming growth factor-beta (TGF-*β*) from the portal circulation in human. 20 liver donors and 20 patients with end-stage liver failure were included in the study. Their blood was collected during liver transplantation from the portal, hepatic, and peripheral vein, and the hepatic artery and cytokines' concentrations were determined. Using the results the mathematical model of cytokine elimination by the liver was developed. In donors significantly lower levels of IL-6, TNF-*α*, HGF, and TGF-*β* were detected in portal blood compared to hepatic vein. In patients with cirrhosis there were no significant differences of IL-6, TNF-*α*, and TGF-*β* levels between portal and hepatic veins. Significantly higher level of HGF in hepatic compared to portal vein was observed. In healthy liver elimination of the cytokines prevailed over their synthesis, as reflected by the positive values of the elimination ratios. In the cirrhotic liver elimination ratios of Il-6, HGF, and TGF-*β* were negative indicating the prevalence of intrahepatic synthesis of cytokines over their removal.

## 1. Introduction

The liver has the unique vasculature involving hepatic artery and vein as well as portal vein. Its strategic location between the digestive system and systemic circulation, as well as the unique metabolic activity in terms of the processes of degradation and synthesis, makes the liver a key organ involved in maintaining homeostasis. Approximately 70% of blood flows into the liver by the portal vein collecting blood from splenic and superior mesenteric vein. The remaining 30% of the blood supply comes from hepatic artery being one of three branches of the celiac trunk. The blood of the portal vein draining the gastrointestinal tract and its rich lymphoid tissue called gut associated lymphoid tissue (GALT) carries significant amounts of various substances consisting of toxins, external antigens, inflammatory mediators, cytokines, and growth factors. Being responsible for providing the first line of defense against the variety of antigens and substances the liver has created its own system of immune response that is both independent and integrated into the immunologic system of the human body. In the course of liver cirrhosis and failure, many pathological phenomena take place, including abnormalities of the gut microflora and intestinal barrier, GALT activation, and endotoxemia. It results in an increase of blood concentrations of proinflammatory molecules such as interleukin-6 (Il-6) and tumor necrosis factor-alpha (TNF-*α*) [1]. It seems that abnormalities affect not only the synthesis of inflammatory mediators by the liver, but also its response to the cytokines and growth factors supplied with the blood [2–4]. The biological effects of those substances depend largely on their concentrations reached locally in the site of action. Therefore, not only the system regulating their production is important, but also organs which contribute to their elimination.

The purpose of the study was to examine the participation of liver in removing growth factors and proinflammatory cytokines from the portal circulation in human. It was achieved by analyzing changes of the concentrations of selected cytokines and growth factors (TNF-*α*, IL-6, HGF, and TGF-*β*) in the blood from portal vein, hepatic artery and vein, and peripheral vein. We analyzed separately two subgroups of subjects according to their liver function: with healthy liver (liver donors) and with liver insufficiency (liver graft recipients). On the basis of our results we elaborated a mathematical model of the hepatic elimination of cytokines and proinflammatory factors.

## 2. Materials and Methods

The study included 20 liver transplant recipients with liver cirrhosis (LC) and 20 deceased liver donors with no liver cirrhosis (NLC) of both sexes. LC patients have liver cirrhosis (Child-Pugh class B and C), negative neoplasm medical history, and glucocorticosteroid treatment. NLC subgroup consisted of patients hemodynamically stable with a negative medical history for liver diseases, chronic inflammatory diseases, neoplasms, diabetes mellitus, connective tissue diseases, and amyloidosis and with negative serology for HCV, HBV, and HIV infection. Liver function and injury parameters in this group were in the normal range (Table 1). In accordance with guidelines of national Center for Organization and Coordination of Transplantation potential donors of liver transplant are carefully evaluated for good liver function and absence of liver disease before procurement and transplantation procedure can proceed.

The demographic and clinical data are presented in Table 1. The study protocol was approved by Bioethical Committee of the Medical University of Warsaw and all study procedures were performed in accordance with the ethical standards of the Declaration of Helsinki and its later amendments. Before any study procedure all liver cirrhosis patients gave their informed consent. It was not possible to obtain informed consent from the deceased donor group.

Blood samples from portal vein, hepatic vein, peripheral vein, and hepatic artery were withdrawn at the beginning of liver procurement surgery from liver donors and during liver transplantation procedure from liver graft recipients at the time of liver explantation into EDTA tubes. Blood samples from each subject were collected simultaneously over 10–20 minutes. Samples were immediately placed on ice and centrifuged at 2000 g for 10 minutes and then plasma was frozen and stored at −70°C until further analyzes which were performed within three months after collection. The sandwich ELISA (enzyme-linked immunosorbent assay) method using commercially available tests (Quantikine ELISA Kit, R&D Systems, Minneapolis, USA) was applied. The absorbance measurements were performed with a use of the photometer for microplates Elx 800 by Bio-Tek Instruments (USA). The absorbance values were read for the wavelength of *λ* = 450 nm with Λ correction 540 or 570 nm. The absorbance was converted into concentration units based upon a standard curve.

The Kolmogorov-Smirnov test was used to assess variables' normality. Since the data have nondistribution, we have applied the nonparameter techniques. Independent groups were compared using the Mann-Whitney *U* test for continuous variables and dependent groups were compared with Wilcoxon test. The correlations were analyzed using the Tau-Kendall ratio. A *P* value of <0.05 was considered as significant.

## 3. Results

### 3.1. Interleukin-6 (Il-6)

In the NLC subgroup a statistically significant difference of IL-6 between the portal vein (PV) and hepatic vein (HV) was observed (0.37 versus 0.22 ng/mL, *P* = 0.02). We observed also a trend towards lower Il-6 concentrations in the peripheral vein (PhV) than PV (0.28 versus 0.37 ng/mL, *P* = 0.061). In LC recipients lower Il-6 concentrations were found in blood from hepatic artery (HA) than from HV and PV (0.28 versus 0.39 and 0.39 ng/mL, *P* = 0.016 and *P* = 0.01, resp.).

### 3.2. Tumor Necrosis Factor-Alpha (TNF-*α*)

In the liver donors higher TNF-*α* concentrations in the PV compared to HV and PhV were observed (11.6 versus 6.3 and 6.9 ng/mL, *P* = 0.03 and *P* = 0.041, respectively). There were no statistically significant differences of TNF-*α* levels in LTX patients. The comparison of NLC and LC subgroups revealed lower TNF-*α* concentrations in HV of NLC patients (6.3 versus 9.56 ng/mL, *P* = 0.034).

### 3.3. Hepatic Growth Factor (HGF)

We observed lower HGF levels in HV versus PV in the graft donors (1.44 versus 1.88 ng/mL, *P* = 0.016). Conversely, HGF concentrations were higher in HV blood than in PV in liver transplant recipients (3.54 versus 2.23 ng/mL, *P* = 0.004). In the hepatic vein we observed lower HGF levels in comparison to PhV (1.44 versus 1.74 ng/mL, *P* = 0.047). There was also a trend towards lower HGF concentrations in HA than in HV (2.3 versus 3.54 ng/mL, *P* = 0.005). While comparing HGF concentrations in both groups, we have found higher levels of this cytokine in the hepatic vein of LC recipients (3.54 versus 1.44 ng/mL, *P* = 0.00007) and lower levels of HGF in PhV of NLC (1.74 versus 2.92 ng/mL, *P* = 0.011).

### 3.4. Transforming Growth Factor-Beta (TGF-*β*)

**Figure 1:**
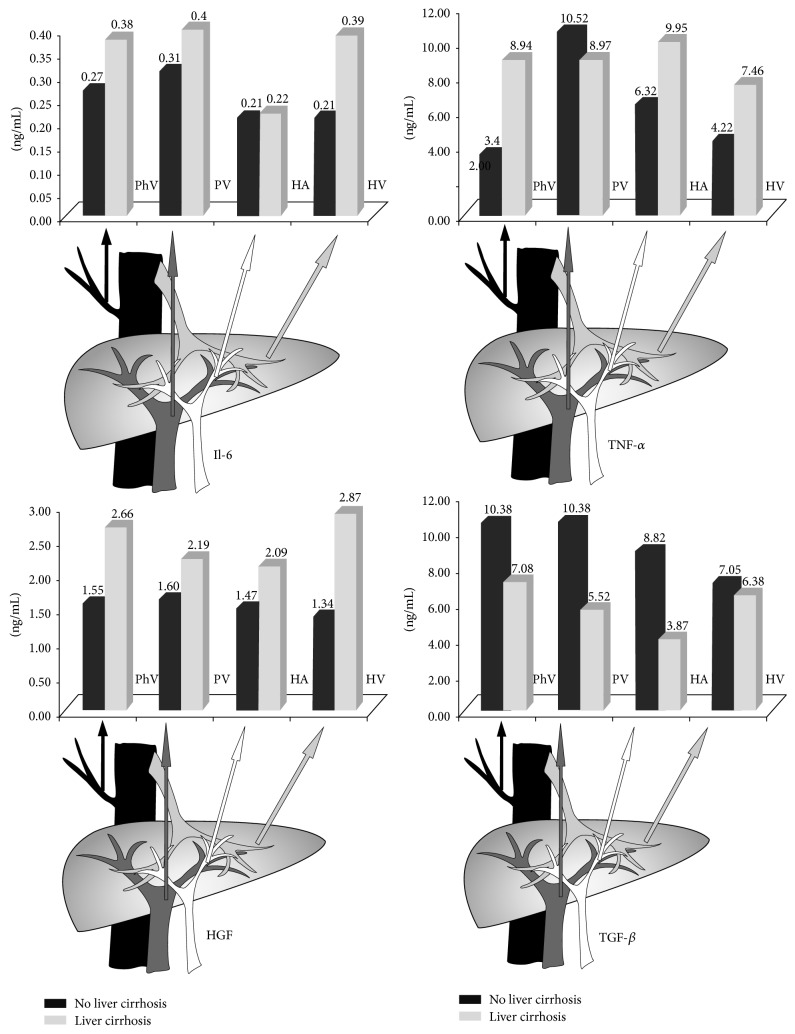
Schematic representation of the concentrations of analyzed cytokines in the blood of peripheral vein (PhV), portal vein (PV), hepatic artery (HA), and hepatic vein (HV). Il-6: interleukin-6, TNF-*α*: tumor necrosis factor alpha, HGF: hepatic growth factor, and TGF-*β*: transforming growth factor beta.

In liver donors we observed lower TGF-*β* levels in the hepatic vein compared to the portal vein and peripheral vein (7.95 versus 12.55 and 11.96 ng/mL, *P* = 0.015 and *P* = 0.049, resp.). There were no statistically significant differences of TGF-*β* levels in LC patients. Lower TGF-*β* concentrations in the blood from AH were observed in liver transplant recipients compared to NLC patients (4.99 versus 9.88 ng/mL, *P* = 0.029). There was also a trend toward lower in portal vein TGF-*β* levels in this LC patient (8.07 versus 12.55 ng/mL, *P* = 0.08). The results of all measurements are summarized in Figure 1.

### 3.5. Mathematic Model of Cytokine Elimination in the Liver

Using the obtained results, the mathematical model of cytokine elimination was developed. The cytokines delivered to the liver via hepatic artery as well as in-hepatic synthesis and degradation were taken into account. Based on the phenomenon of the first pass effect known in biopharmaceutics and pharmacokinetics the extraction ratio (ER) formula that considered the above-mentioned processes was elaborated, as follows: ER = (*C*_in_ − *C*_out_)/*C*_in_, where *C*_in_ = concentration of a substance entering the liver and *C*_out_ = concentration of a substance leaving the liver. In the next step the equation was enriched by adding variables reflecting the synthesis and degradation of cytokines by the liver: *C*_syn_ = concentration of a substance synthesized in the liver, *C*_in/deg_ = concentration of a substance entering the liver and then degraded, and *C*_syn/deg_ = concentration of a substance synthesized in the liver and then degraded, to yield the following equation for calculation of the liver elimination ratio (LER):

(1) LERCin−Cin+Csyn−Cin/deg−Csyn/degCin=−Csyn+Cin/deg+Csyn/degCin.

Assuming that extraction ratio (ER) is equal to the liver elimination ratio (LER) we obtain the following formula: *C*_in_ − *C*_out_ = −*C*_syn_ + *C*_in/deg_ + *C*_syn/deg_. Negative LER values reflect the predominance of cytokine synthesis over their degradation. If the degradation predominates the synthesis the elimination ratio is positive. Because the LER formula comprises more than one unknown variable, to calculate the rate of hepatic elimination we have used the ER equation.

### 3.6. Elimination Ratio of Cytokines by the Liver

The elimination ratio (LER) was calculated according to the formula given above for each of the cytokines: Il-6, HGF, TNF-*α*, and TGF-*β*. In the healthy liver the elimination of analyzed cytokines surpassed their in-liver synthesis based on the positive value of the elimination ratios: 0.4, 0.2, 0.4, and 0.3, respectively. In liver transplant recipients (liver failure), we observed negative values of WE ratios for Il-6, HGF, and TGF-*β* (−0.1, −0.5, and −0.2, respectively). The WE ratio of TNF-*α* had a positive value (0.1).

## 4. Discussion

The presented results have confirmed the important role of the liver in the development and course of immune response through the synthesis and removal of proinflammatory cytokines and growth factors. In liver graft donors, we have observed significantly lower concentrations of all studied cytokines in the hepatic vein than in the portal vein, which suggests a significant role of the liver in the removal of these molecules from systemic circulation. Noteworthy is the absence of such differences in liver transplant recipients. Moreover, in patients with liver failure the concentrations of HGF in the blood leaving the liver were higher than in the portal vein, which may indicate its synthesis in the setting of hepatic insufficiency.

We have shown that liver cirrhosis and failure are associated with not only impairment of detoxification and synthetic and metabolic processes, but also alterations and even increased synthesis of some proinflammatory molecules. The role of the liver as one of the major sites of cytokines production has been widely acknowledged in the literature [5–10]. Liver function abnormalities in the course of its cirrhosis and insufficiency may have a direct impact on the circulating cytokines and growth factors and ultimately affect the immune system function [11]. Most of the available studies on the removal of cytokines and growth factors by the liver were carried out in animal models with the use of exogenous labeled inflammatory mediators and not considering their intrahepatic synthesis and degradation [12–19]. In the present study, thanks to the simultaneous assessment of the concentrations of the cytokines in the blood from the hepatic artery and vein and the portal and peripheral vein, analysis of intrahepatic processes was possible. In addition, the elaborated equation for the elimination ratio calculation allowed for estimation of the intrahepatic processes and the role of the liver in the synthesis and elimination of studied cytokines. Applying the elimination ratio for the evaluation of hepatic cytokine removal allowed us to make obtained results independent of the possible impact of variability in cytokine production resulting from comorbidities in liver transplant donor and recipients, causing death of liver graft donors.

We observed that in the healthy liver the elimination of Il-6 predominates, which is consistent with the results of studies in animal models. Castell et al. described the plasma clearance of labeled Il-6 in rat model [12, 20]. In the case of liver failure and cirrhosis the presence of elevated levels cytokines and growth factors were also demonstrated [21–28]. It may result both from a limited degradation and enhanced intrahepatic synthesis of these molecules. In our study this hypothesis was confirmed by the negative WE ratio values of Il-6, HGF, and TGF-*β* in liver transplant recipients that were lower in comparison to liver graft donors. Other authors observed higher Il-6 and TNF-*α* concentrations in patients with liver cirrhosis compared with healthy volunteers [1]. The authors pointed at the existence of the correlation between cytokines' levels and the severity of liver cirrhosis. The negative value of Il-6 elimination ratio in our group of liver graft recipients may result from both impaired degradation and increased intrahepatic synthesis in response to increased concentrations of nondegraded toxins. Significantly lower TNF-*α* concentrations in the hepatic vein compared to the portal vein found only in the NLC prove an important role of the healthy liver in the elimination of this molecule. The elimination ratio of TNF-*α* was comparable to LER for Il-6 in NLC. In the case of liver failure we did not observe negative WE ratios of TNF-*α* which may indicate a lack of its increased intrahepatic synthesis. In the case of HGF, its rate of elimination by healthy liver was similar as observed for Il-6 and TNF-*α*. However, in liver transplant recipients higher HGF levels were observed in hepatic vein than in portal vein. The finding of significantly higher HGF concentrations in the hepatic and peripheral veins of LC patients compared to the liver graft donors may indicate that in the latter group the liver mainly eliminates the growth factor, while in NLC patients its intrahepatic synthesis predominates the rate of elimination. Liu et al. showed that in the first pass the liver eliminates approximately 26% of labeled HGF and in the early stage 70%, and what is interesting is the fact that the molecule is also taken up by the adrenal glands, spleen, kidneys, and lungs [29]. Studies in both humans and animals showed elevated levels of HGF in the systemic circulation associated with different liver pathologies [19, 30–38]. Rudi et al. suggested, however, that increased HGF levels are associated rather with liver failure than with hepatocytes' regeneration in the course of liver cirrhosis [37]. The hypothesis that the pathological processes taking place in the liver increase intrahepatic HGF synthesis is confirmed by our finding of strongly negative LER ratio in liver transplant recipients. Lower TGF-*β* concentration in the hepatic vein compared to portal and peripheral veins in NLC evidence its hepatic elimination and participation of other peripheral organs in its removal. A significant role of the liver and a smaller impact of other organs in removing of TGF-*β* were also shown by other researchers [39, 40].

The weaknesses of our study include mainly small size of the study groups. This was caused by the pilot nature of the project and also by the logistic difficulties associated with obtaining blood samples in such specific clinical circumstances.

In conclusion, the differences in Il-6, TNF-*α*, HGF, and TGF-*β* observed in liver donors between the portal and hepatic veins seem to result from their removal by healthy liver, which is absent in liver graft recipients with hepatic insufficiency. The negative elimination ratios of Il-6, HGF, and TGF-*β* indicate the prevalence of their intrahepatic synthesis in liver failure. Higher concentration of HGF in the hepatic vein and its strongly negative elimination ratio suggest that this growth factor may be intensively synthesized in the milieu of liver failure. To the best of our knowledge it is one of the few human studies simultaneously evaluating concentrations of Il-6, TNF-*α*, HGF, and TGF-*β* in the hepatic artery and vein and in the portal and peripheral veins in the setting of good and poor liver functions. We present also a unique equation aimed at assessment of the role of the liver in the removal and synthesis of a given substance.

## Figures and Tables

**Table 1:** Demographic and clinical data of the study group. F: female, M: male, and SD: standard deviation.

Parameter	No liver cirrhosis (NLC)	Liver cirrhosis (LC)
Sex F/M [*n* (%)]	5 (25)/15 (75)	11 (55)/9 (45)
Age [years (SD)]	35.3 (12.7)	50.1 (8.5)
Cause of death [*n* (%)]		
Craniocerebral trauma	9 (45)	
Hemorrhagic stroke	9 (45)	
Subarachnoid hemorrhage	2 (10)	
Child-Pugh class [*n* (%)]		
B		13 (65)
C		7 (35)
Cause of liver failure [*n* (%)]		
Unknown		6 (30)
Primary biliary cirrhosis (PBC)		3 (15)
Alcoholic liver disease (ALD)		3 (15)
Hepatitis C cirrhosis (HCV)		3 (15)
HBV + HCV cirrhosis		2 (10)
Primary sclerosing cholangitis (PSC)		1 (5)
Autoimmune hepatitis (AIH)		1 (5)
AIH + HBV		1 (5)
AlAT (U/L) [mean (SD/range)]	29.2 (16.1)	458 (11–6766)
GGTP (U/L) [mean (SD/range)]	24.1 (17.1)	188.5 (38–476)
INR [mean (SD/range)]	1.2 (0.2)	1.4 (0.86–4.0)
Creatinine (mg/mL) [mean (SD/range)]	0.9 (0.24)	1.0 (0.3)
Interleukin-6 (Il-6) [median (range) ng/ml]		
Portal vein	0.31 (0.12–0.90)	0.40 (0.21–0.62)
Hepatic vein	0.21 (0.02–0.44)	0.39 (0.19–0.88)
Hepatic artery	0.21 (0.01–1.07)	0.22 (0.01–0.60)
Peripheral vein	0.27 (0.02–0.74)	0.38 (0.04–0.55)
Tumor necrosis factor-alpha (TNF-*α*) [median (range) ng/ml]		
Portal vein (PV)	10.52 (2.98–35.81)	8.97 (1.98–19.68)
Hepatic vein (HV)	4.22 (0.48–22.36)	7.46 (2.47–22.36)
Hepatic artery (HA)	6.32 (0.59–36.11)	9.95 (1.48–45.27)
Peripheral vein (PhV)	3.40 (0.48–38.01)	8.94 (0.24–21.59)
Hepatocyte growth factor (HGF) [median (range) ng/ml]		
Portal vein (PV)	1.60 (1.00–6.18)	2.19 (0.97–4.02)
Hepatic vein (HV)	1.34 (0.71–2.92)	2.87 (1.12–8.94)
Hepatic artery (HA)	1.47 (0.92–4.38)	2.09 (0.72–4.50)
Peripheral vein (PhV)	1.55 (0.52–5.32)	2.66 (0.99–5.96)
Transforming growth factor-beta (TGF-*β*) [median (range) ng/ml]		
Portal vein (PV)	10.38 (3.69–28.29)	5.52 (2.96–24.60)
Hepatic vein (HV)	7.05 (3.42–15.82)	6.38 (4.85–18.78)
Hepatic artery (HA)	8.82 (2.68–23.24)	3.87 (1.52–11.83)
Peripheral vein (PhV)	10.38 (1.21–26.08)	7.08 (1.91–21.19)
